# Influence of Migratory Strategy, Group Size, and Environmental Conditions on the Movements of Caribou in Eastern Alaska

**DOI:** 10.3390/ani15101453

**Published:** 2025-05-17

**Authors:** Kyle Joly

**Affiliations:** Gates of the Arctic National Park and Preserve, Yukon-Charley Rivers National Preserve, National Park Service, Fairbanks, AK 99709, USA; kyle_joly@nps.gov

**Keywords:** collaring impacts, density, migration, Nelchina, *Rangifer tarandus*, snow, temperature

## Abstract

Migration is a diverse behavior exhibited by a wide array of organisms. Variability in the type of movements is rooted in their purpose, environmental factors, demographics, and individual physiological condition. The ability of caribou (*Rangifer tarandus granti*) to efficiently move long distances and have a high degree of plasticity in their movements allows them to respond and be resilient to dynamic and dramatic differences in environmental conditions. I used 88 collared, sympatric, adult, female, barren-ground Nelchina Caribou Herd caribou in east-central Alaska to assess their migratory strategy (as indexed by the distance between winter and summer ranges) and how this might affect their movements. Employing 41,682 movement vectors from 39 of these individuals equipped with GPS collars, I compared the annual and monthly movements of caribou that were found on different winter ranges. Distances between winter and summer ranges for individual caribou were correlated with their annual movement, but not for caribou that wintered within the same area. As expected, caribou with the greatest distance between their winter and summer ranges (300 km) traveled the most annually (2132 km/year), whereas caribou with the shortest distance between ranges (71 km) traveled the least annually (1368 km/year). However, caribou that migrated the furthest exhibited greater movement rates in all non-migratory summer months and most non-migratory winter months, as well as during migration. Movement rates were the greatest in summer, peaking in July, regardless of where caribou wintered. During the winter months, movement rates were similar among caribou found on different winter ranges and decreased over the winter, reaching minimums in January-March. Caribou that migrated the shortest distance and had lower movement rates tended to be found in smaller groups in summer. The connection between group size and movement rates may be a function of competition or a small-scale example of the larger-scale phenomenon of range expansion of large herds. Environmental factors, such as snow depth and temperature, were also correlated (negatively and positively, respectively) with caribou movement rates. Survival was not significantly different for caribou utilizing different winter ranges, which implies that the benefits of this long-distance migration can be offset by its costs. A more detailed understanding of the drivers and variability of caribou movement should help improve the management of this declining species.

## Simple Summary

Many factors affect the movements of migratory animals. The objective of this study was to assess if migratory strategy (whether individuals migrated to distant winter ranges or remained close to their summer range), the number of other individuals present nearby (group size), and environmental conditions, like snow depth and temperature, affected the movements of caribou. Migratory caribou moved more than resident caribou even during non-migratory months. Caribou found in larger groups also had greater movement rates. Warmer temperatures and shallower snow were associated with greater movement rates. A better understanding of what drives movements of caribou will help improve the management of this globally declining species.

## 1. Introduction

Research on migratory behavior, movement ecology, and their variability enhances our knowledge of ungulate energetics, susceptibility to predation, fitness, and, ultimately, management and conservation [1,2,3]. Migration is selected for if its benefits outweigh its costs [4,5]. The benefits of migration may include improved access to forage, mates, reduced predation risk, and/or favorable environmental conditions. Costs may include greater energetic expenditures, competition with conspecifics, or increased predation risk.

Barren-ground caribou (*Rangifer tarandus*) are known for their extensive movements and migrations. These migrations, the longest of any terrestrial mammal [6], take them to and from their calving grounds and winter ranges. However, not all barren-ground caribou exhibit typical migration patterns [7]. Migration distances vary among herds; some herds exhibit partial migration, and some individuals exhibit facultative migration. Given their low fidelity to winter ranges, migratory distances vary even within herds and across years [7,8].

Movement rates are associated with migratory strategy, animal density, and environmental conditions. Given increased distances between seasonal ranges, longer-distance migrants travel farther than shorter-distance migrants and resident individuals. However, movement rates can vary among individuals, employing different migratory strategies even during non-migratory periods [9]. This has been attributed to migratory individuals adopting a strategy to maximize energy intake, whereas resident individuals attempt to minimize energy expenditures [9]. Although density (group or band size) may influence caribou movements [9,10], the relationship is still not well documented. Temperatures can also influence caribou movement rates, with high summer temperatures being associated with high levels of insect harassment and the greatest movement rates of the year [11,12,13,14]. The effects of winter temperature on caribou movement rates are less pronounced [9]. While caribou are highly efficient walkers, deep snow can increase the energetic costs of movement and slow movement rates [11,15,16]. The plasticity in migratory strategies and movements exhibited by caribou allows them to be resilient to the wide array of environmental conditions that they face over the range of their circumpolar distribution seasonally, annually, and as climate fluctuates.

Movement rates have been estimated for woodland (*R. t. caribou*) [17] and Alaska caribou (*R. t. granti*) using activity budgets [18], direct observation [13,19], pre-GPS satellite telemetry [17,20,21,22], and GPS telemetry [14,23,24]. Even when the same subspecies and techniques are used, sampling intervals can vary, see [17,20,21,22], which can greatly influence estimates [24,25]. The use of different subspecies (barren-ground versus woodland), sampling techniques, and sampling intervals makes inter-study comparisons difficult. Teasing apart the various influences on individual movement rates can be achieved by studying a single herd of caribou fitted with collars with the same sampling scheme.

My objectives were to quantify the year-round movements of caribou and to determine if migratory strategies (i.e., selection of wintering area), density (as indexed by group size), and environmental conditions were associated with movement rates. I predicted that different migratory strategies would be employed and that movement rates would vary among caribou utilizing these different strategies, time of year, group size, and environmental factors. Finally, I assessed the cost of migratory strategy in terms of survival.

## 2. Materials and Methods

### 2.1. Study Population and Area

**Figure 1:**
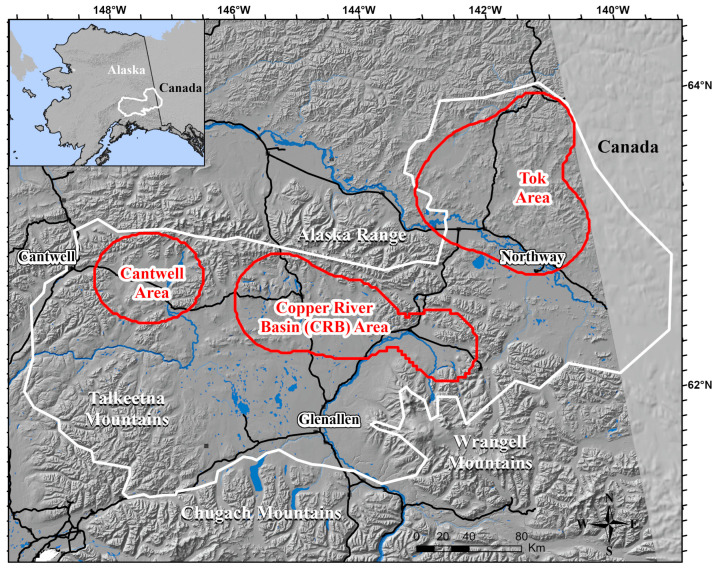
Range of the Nelchina Caribou Herd (white polygon), east-central Alaska, October 1999–September 2002. Wintering areas (red polygons) are based on 95% kernel analysis from 1999–2001.

From 1999–2003, the Nelchina Caribou Herd (NCH) numbered around 33,000 animals [26], the majority of which calve on the eastern flanks of the Talkeetna Mountains. Post-calving aggregations of several thousand individuals form by July [27]. The NCH normally remains in the Nelchina Basin until September, when fall migration commences. For a few decades, the majority of the herd has wintered in the boreal forests on the north side of the Alaska Range, near the USA-Canada border [28] (Figure 1). Before the 1990’s, the herd wintered south of the Alaska Range within the boreal forest of the Copper River Basin (CRB). They stayed in the CRB during the winter of 2001–2002. The causes of these changes in distribution are unknown, but is likely a combination of biotic and abiotic factors. Interestingly, some members of the NCH do not migrate in the fall with the main herd; their winter range is centered on the northern Talkeetna Mountains (Figure 1). They do, however, show extensive overlap of summer range with the majority of the herd. Some have referred to these caribou as the Upper Susitna Herd [29]. Starting in April, the entire herd begins its trek back to the calving grounds in the southern Talkeetna Mountains. Caribou herds have traditionally been defined by where they calve [30].

The NCH ranges from east of the USA-Canada border to north of the Alaska Highway, to the Wrangell Mountains, to the east side of the Talkeetna Mountains, and to the town of Cantwell (Figure 1). Elevation ranges from 300 m to nearly 5000 m, although the caribou tend to be found below 2000 m. Vegetative cover is highly associated with elevation. Muskegs and tussock tundra are common in the low-lying areas. Black spruce (*Picea mariana*) is the dominant tree species, though aspen (*Populus tremuloides*), birch (*Betula papyrifera*), and white spruce (*Picea glauca*) stands can be found. Shrubs (*Alnus* spp., *Betula* spp.) give way to alpine communities at higher elevations.

The climate is variable within the herd’s range, though it is entirely within the interior of Alaska (61–64° N, 141–150° W). Summers tend to be cool and wet, especially in the Talkeetna Mountains, where the entire NCH calves and spends the post-calving period. The wintering grounds north of the Alaska Highway tend to be very cold (temperatures reaching −50 °C) and dry. The wintering grounds in the northern Talkeetna Mountains are warmer and wetter, but still relatively cold and dry. The CRB, lying between these two areas, tends to have winter temperatures and snowfall that are intermediate between the two other wintering grounds. The fall of 2001 was the warmest on record (at that time).

### 2.2. Data Collection

Adult female caribou were captured using standard helicopter darting techniques [31,32] beginning in October 1999. The caribou were instrumented with a VHF radio collar, some of which were also equipped with a Global Positioning System (GPS) scheduled to acquire positional data every 7 h. The VHF-only collars were from Telonics, Inc. (Mesa, AZ, USA), Mod-500s, and the GPS collars were Advanced Telemetry Systems (Isanti, MN, USA) with Garmin GPS 25LP receivers. The average location error was 33 m [24]. Every 6 months, until October 2002, the caribou with GPS collars were recaptured and given new collars. During these captures, new caribou were added to the sample, and others had their collars removed and were released. All caribou were radiotracked monthly, using fixed-wing aircraft. Group size, the estimated number of caribou with the instrumented individual, was recorded on these tracking flights [27].

### 2.3. Movements

I used mid-winter (January, if available—<5% were the next closest location, e.g., December or February) locations for each year to assign NCH caribou to different winter ranges by creating a 95% fixed kernel estimator [33,34] using the least squares cross validation (LSCV) smoothing parameter. Using the entire dataset, the procedure produced three distinct wintering areas: Cantwell, CRB, and Tok (Figure 1). Caribou with mid-winter locations falling outside one of the three kernels were assigned to the group of their nearest neighbor. I created 95% kernel estimators to delineate a summer range for each winter range based on mid-summer (July, if available—<5% were the next closest location, e.g., June or August) locations. I calculated the distance between mid-winter locations and the following mid-summer location. I used analysis of variance (ANOVA) [35] to determine if there were significant differences in the distance between mid-winter locations and mid-summer locations. I also analyzed individual fidelity to the winter area by following changes in the wintering area annually.

GPS data were downloaded from the collars and imported into an ArcGIS Pro (Esri, Redlands, CA, USA) Geographic Information System (GIS). Greenwich Mean Time was converted to Alaska Standard Time. Movement vectors, the distances between successive relocations, for each individual were determined for GPS-collared individuals. For those caribou that had an entire year’s worth of data, a minimum annual distance traveled was calculated. I defined minimum annual distance as the summation of all movement vectors for a given study year (generally October 1 to September 30 of the following calendar year). For caribou with multiple years of data, minimum annual movement was calculated for each year. Minimum annual movement was not calculated unless the caribou had at least 750 relocations during the year. I tested for correlations between the distance between mid-winter and mid-summer locations and the minimum annual distance traveled. I used ANOVA [35] to determine if there were significant differences in minimum annual distance traveled among the migratory groups.

The lengths of movement vectors were divided by the number of hours between relocations in order to determine an hourly movement rate. I then calculated the average hourly movement rate for each individual by month. I used ANOVA to analyze potential differences in movement rates by month among groups. Caribou locations in the three different winter areas were assigned climatic data (average temperature, departure from average temperature (absolute value to eliminate negative terms)), and snow depth from the nearest of three sites (Cantwell, Alaska, for the Cantwell area, Glenallen, Alaska, for the CRB area, and Northway, Alaska, for the Tok area, all collected by the National Atmospheric and Oceanic Administration’s National Climatic Data Center, https://www.ncei.noaa.gov/cdo-web/; accessed 19 March 2025). Snow depth data were only available for February through May.

The hourly movement rate differed by the winter area used throughout the year (see Results); therefore, I not only analyzed the data on an annual basis, but also just during the summer (June, July, August, and September), winter (December, January, February, and March), migration (April, May, October, and November), months with snow data (February, March, April, and May), and non-migratory months with snow data (February and March). I used logistic regression to analyze the relative effects of environmental variables and group size on the hourly movement rate during these various sampling periods. I chose models based on the hypothesized importance to caribou ecology for the specific sampling period (see Results. Models were ranked and selected using Akaike’s Information Criteria for small sample sizes (AIC*_C_*) and Akaike weights (*w_i_*) [36].

I determined the angular deviation between consecutive movement vectors. This provided an index of how linear the movements of different individuals were. I then used an ANOVA to determine if the linearity of movements varied among caribou from the various wintering areas during the non-migratory summer months (May through September).

### 2.4. Survival

I analyzed the survival of caribou using the different winter ranges annually (both VHF and GPS collars). I divided the study into six 6-month periods for known fate survival analysis. I constructed two models to examine variation in monthly survival relative to migratory behavior. The first model examined all individuals as one group, and the second model divided the caribou based on their winter range distribution (Cantwell, Tok, CRB; see above). I used Akaike’s Information Criterion, adjusted for the small sample size (AIC*_C_*) [36,37], to compare these models. Survival rate analyses were conducted with the MARK software package (http://www.phidot.org/software/mark/docs/index.html; accessed 19 March 2025) [37]. Similarly, I compared survival between VHF- and GPS-collared caribou. I censored capture mortalities.

A significant difference was detected (see Results) between the survival of VHF- and GPS-collared caribou. Because of this difference between groups, VHF and GPS data should not be pooled to analyze differences among groups. I, therefore, re-analyzed survival for the different winter ranges of VHF- and GPS-collared caribou using the aforementioned technique. The first model examined all individuals as one group and the second model divided the caribou into six subsets based on their winter range distribution and collar type (VHF or GPS).

## 3. Results

A total of 88 adult female caribou were captured and instrumented with VHF collars. There were 8 capture-related mortalities across 144 captures. Of the 88 with VHF collars, 39 also had GPS units that yielded 41,682 movement vectors from October 1999 to September 2002. The average fix rate (percent of successful relocation attempts) for all GPS units was 77.5% (range 19.6–99.8%). Kernel analysis indicated that the 88 caribou migrated to 3 distinct winter ranges during the study period (Figure 1). Three winter locations were located south of the CRB area kernel, and those caribou were assigned to the CRB. The mean distance between mid-winter and mid-summer locations was four times as great for the Tok area (300 km, range 220–384 km, *n* = 96) and twice as great for the CRB area (146 km, range 80–248 km, *n* = 38) than the Cantwell area (71 km, range 10–123 km, *n* = 17) caribou. These differences were significant (*F* _2, 148_ = 551.36, *p* < 0.01). The summer ranges of the caribou that wintered in the Tok and CRB areas were sympatric with the summer range of the caribou that wintered in the Cantwell area, with 96% and 97% of their summer ranges contained within the summer range of the Cantwell area caribou, respectively.

NCH caribou showed little affinity to a specific winter range during the study period. Although relatively few (11.4%) caribou (4 of 35) switched winter ranges between the first and second years of the study, 79.4% (27 of 34) switched winter ranges between the second and third years. Of these, 77.8% had wintered north of the Alaska Highway (Tok area) but spent the third winter (2001–2002) within the CRB area, south of the Alaska Range. The fall of 2001 was the warmest ever recorded in the state (at that time). Of the 32 Tok caribou I had instrumented in the second year of the study, only 12.5% migrated back to the same region for the third winter. My multi-year data for Cantwell and CRB were limited (*n* = 5, 3, respectively) but revealed that 60.0% and 33.3% of these groups, respectively, migrated to a different winter range in subsequent years.

Group sizes were greatest in July, diminished in August, and were low in December and January for caribou that wintered in the different areas (Table 1). Group sizes for all caribou were also small in May during calving, with large increases seen in June, typical of post-calving aggregations. For other months, changes in group size varied by where caribou previously overwintered (Table 1).

**Figure 2:**
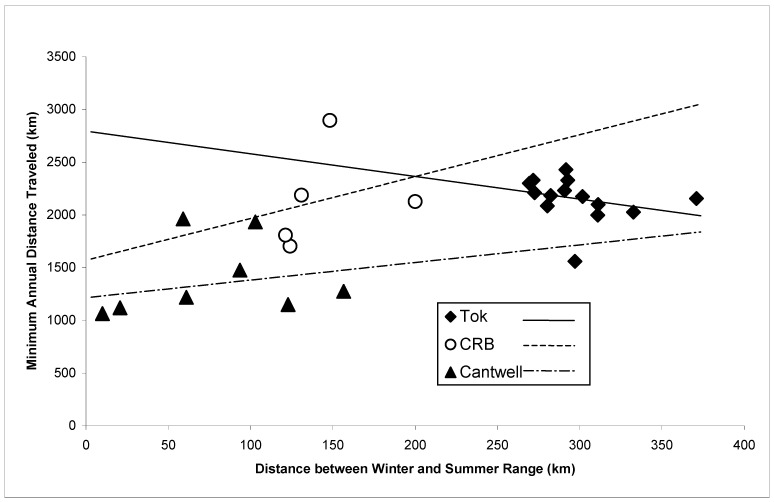
Comparison of distance between winter and summer ranges and minimum annual distance traveled by individual caribou using different Nelchina Caribou Herd wintering areas, east-central Alaska, October 1999–September 2002.

The minimum annual distance traveled was not significantly different among years for any of the different winter ranges (Cantwell, *F* _1, 7_ = 1.96, *p* = 0.20; Tok *F* _2, 14_ = 1.58, *p* = 0.24; CRB, *F* _1, 5_ = 0.00, *p* = 0.96); therefore, I pooled the data across years. The mean minimum annual distance traveled by the caribou that wintered in the Tok area (2132 km/year; range 1559–2429 km/year) or CRB (2120 km/year; range 1704–2897 km/year) was significantly (*F* _2, 30_ = 22.98, *p* < 0.01) greater than the distance traveled by caribou that wintered in the Cantwell area (1368 km/year; range 1063–1962 km) but not from each other (*p* > 0.05). The distance between mid-winter and mid-summer locations was positively correlated with the minimum annual distance traveled (*R*^2^ = 37.3, *F* = 14.84, *d.f.* = 26, *p* < 0.01) when data were pooled. However, the two metrics were not correlated if the caribou that wintered in any one area were analyzed separately (Figure 2; Cantwell, *R*^2^ = 3.9, *F* = 0.24, *d.f.* = 7, *p* = 0.64; Tok, *R*^2^ = 6.6, *F* = 0.85, *d.f.* = 13, *p* = 0.37; CRB, *R*^2^ = 8.8, *F* = 0.29, *d.f.* = 4, *p* = 0.63). From May to September, the movements of caribou that wintered in the Tok area were more linear than those of caribou that wintered in the Cantwell area (*F* _2, 143_ = 5.06, *p* = 0.03).

**Figure 3:**
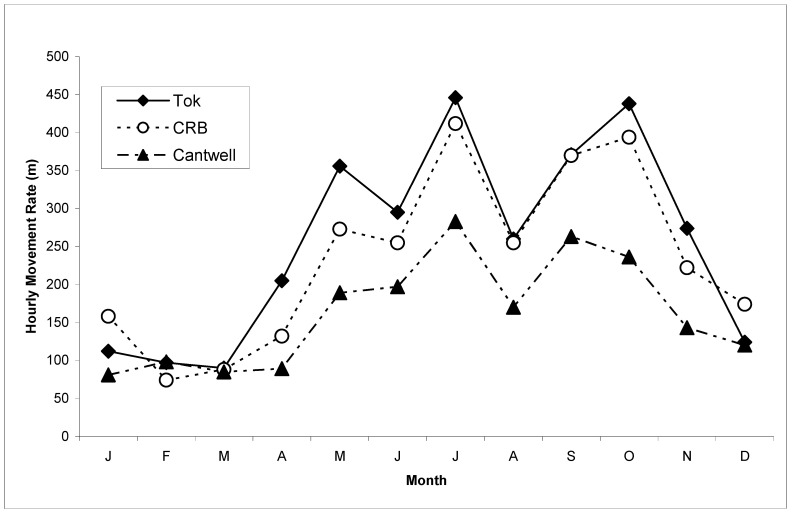
Hourly movement rates by month of caribou that used different Nelchina Caribou Herd wintering areas, east-central Alaska, October 1999–September 2002.

The caribou that wintered in the Tok and CRB areas exhibited greater hourly movement rates than the caribou that wintered in the Cantwell area in every month except for February (Table 2, Figure 3). Hourly movement rates were greatest in July for all caribou groups (446 m/h, Tok; 412 m/h, CRB; 290 m/h Cantwell). The caribou that wintered in the Tok area had their lowest movement rates in March (90 m/h), CRB in February (74 m/h), and Cantwell in January (81 m/h).

Year-round movements were also related to wintering area, average daily temperature, and deviations from normal temperatures (Table 3A). Movement rates were positively related to average temperature but negatively related to deviation from normal temperatures. Group size may also be a factor in the year-round movement of these caribou (Table 3A). Group size was the only factor in the most parsimonious model predicting movements during the summer season (Table 3B). Movement rates were positively related to group size. During the winter months, average daily temperature was the only factor to enter the model (Table 3C). Caribou had greater movement rates at lower temperatures; however, winter temperatures were generally mild during the study period. Movements during migratory months were related to deviations from normal temperatures (Table 3D). Movement rates during these months were reduced when temperatures were far from normal temperatures. Average temperature and snow depth were related (positively and negatively, respectively) to movement rates from February through May (Table 3E). The results suggest a more complicated model for mid-winter (February and March; Table 3F), but movement rates were still negatively related to snow depth. Dropping the more migratory months of April and May out led to a negative relationship between movement rates and average temperature.

Caribou that wintered in the Cantwell area showed higher survival (84.4%) for the duration of the study versus both the Tok (74.5%) and CRB (79.0%) wintering areas. However, the 95% confidence intervals substantially overlapped (Cantwell 39.2–97.9%, Tok 58.8–85.7%, CRB 51.4–93.1%). The survival model assigning all caribou as the same group was a better model than distinguishing the caribou by migratory group (Table 4A). GPS-collared caribou had lower annual survival (97.8%) than VHF-collared caribou (99.2%). The survival model assigning GPS- and VHF-collared caribou to different groups was a better model than grouping all the caribou together (Table 4B).

When parsing by collar type (VHF and GPS), caribou that wintered in the Cantwell area showed higher survival than the caribou that wintered in the Tok area (100% versus 97.0%, 94.1% versus 86.0%, respectively). However, the 95% confidence intervals substantially overlapped (Cantwell VHF 100–100%, Tok VHF 93.0–98.7%, Cantwell GPS 68.0–99.2%, Tok GPS 72.2–93.6%), resulting in no statistical differences between them. GPS-collared caribou had lower survival than VHF-collared caribou for all three wintering areas (Cantwell 94.1% versus 100%, Tok 86.0% versus 97.0%, CRB 95.2% versus 96.6%). The survival model assigning all caribou as a single group showed a better fit than the model distinguishing the caribou by wintering area and collar type based on AIC scores (Table 4C). The lack of significance may be influenced by small sample sizes.

## 4. Discussion

Migration, as a life history strategy, is selected for if the benefits outweigh the costs [38]. Variability in migration strategies can occur between and within species, populations, and individuals [7,39,40,41,42]. Migration may be beneficial if it improves access to abundant high-quality food, less predation pressure, or both [4]. Environmental factors are also critically important, as studies have revealed that the proportion of individuals migrating increases with winter severity and more (temporally) variable habitats [41]. Plasticity and variability, at both the species and individual levels, in migration and movement strategies may increase their resiliency to detrimental environmental conditions [42].

Barren-ground caribou, found in harsh arctic and subarctic climes, are known for extensive movements and dramatic migrations [6]. Long-distance seasonal migratory behavior, however, is not ubiquitous in this species even within regions ([7,43], this study). NCH caribou have sympatric summer ranges but exhibit various migratory strategies, reaching winter ranges that can be within 10 km or as distant as 384 km away. Migratory strategies varied annually among individuals, as NCH caribou showed little fidelity to their winter ranges from 1999 to 2002. Almost every possible change between winter ranges occurred during the short span of this study. The majority of the herd forgoing the migration to the Tok area was an anomalous event that had not been reported for a decade. It is possible that it was triggered by highly unusual warm conditions seen throughout the summer and fall of 2001.

As expected, minimum annual distance traveled was significantly greater for those caribou migrating to the distant winter range north of the Alaska Highway (Tok area) than for those that did not migrate to the Tok area and stayed in the nearby northern Talkeetna Mountains (Cantwell area) year-round. Although the minimum annual distances traveled reported by Joly et al. [6] for northern caribou were more than double the distances I report here for interior caribou, there are many striking similarities to my results. Fancy et al.’s [20] analyses revealed that members of the Central Arctic Herd (CAH), with their “less distinctive migration patterns between overlapping seasonal ranges”, had minimum annual movements that were only 70% as long as members of the migratory Porcupine Caribou Herd (PCH). The difference was thought to be attributable to herd size [20]. This explanation also has merit for interior Alaska caribou, as the number of NCH caribou that migrate long distances to winter in the Tok area far exceeds the number of caribou that head to the closer Cantwell area for winter. Density is a factor that influences movement patterns ([39], this study).

The differences in the minimum annual distances traveled cannot be entirely attributed to longer distances between winter and summer ranges, as the annual distance moved was not significantly correlated if wintering areas were analyzed separately. Furthermore, caribou that wintered in the Tok and CRB areas had significantly greater hourly movement rates than the caribou that wintered in the Cantwell area from May until September and every winter month except February, which are primarily non-migratory months. The caribou that wintered in the Tok and CRB areas formed large aggregations during the summer months. I hypothesize that the greater movement rates by these caribou during these non-migratory summer months were due to these large aggregations quickly depleting forage within an area, which required the group to move more frequently [39]. This hypothesis is supported by hourly movement rates that were positively correlated with group size and the Tok caribou having more linear movements than the Cantwell caribou during this period. This suggests that the smaller bands of Cantwell caribou were able to remain in an area longer than the Tok caribou. An alternative, yet not mutually exclusive, hypothesis is that the groups with higher summer movement rates are exposed to greater insect harassment [14,44].

The nexus between group size and movement rates also has other implications. Large herds are known to expand their ranges into new territories [30,43,45,46]. If the small-scale movements of large groups are in response to diminished localized forage, then I posit that this phenomenon might be a precursor to the range expansions (large-scale movements) of large herds and potentially of migration itself. At low densities, caribou can be more sedentary not only because of reduced competition for food resources but also because of reduced predation rates [47,48]. At high densities, caribou use migration not only to acquire new food resources but also to move away from predators [48,49]. Low-density herds that increase to high density can switch to a migratory lifestyle [43] and vice versa [30,45]. The former may have been the situation for the NCH when it abandoned its traditional wintering grounds within the CRB for the Tok area as the herd size increased. The relationships among density, migration, range depletion, predation, and life history strategies require further exploration and research.

Fidelity to wintering areas was low. This result agrees with previous studies [7,8]. The caribou that wintered in the Cantwell area typically spend the fall, rut, winter, and spring apart from the rest of the NCH. While caribou herds are defined by where they calve [30], and thus the caribou that winter in the Cantwell area have been considered part of the NCH, their separation during the rut (when genetic flow occurs) and differing ecology warrant further consideration of solely using calving grounds to determine herd identity [50].

Post-calving aggregations are, in part, a tactic to assuage insect harassment. Group sizes were greatest in July, corresponding to the peak of insect harassment. NCH caribou from every wintering area had their greatest movement rates during July, which is also consistent with previous findings [14,20]. Caribou may have greater movement rates in the summer due to increased insect harassment in the areas used by the NCH [12,13,19,51], because they abandon areas of parasitic warble fly (*Hypoderma tarandi*) emergence [52], or increased group size [this study].

During late winter, before the emergence of parasitic insects, caribou movements were positively correlated with temperature and negatively correlated with snow depth. Although caribou are very well adapted to the harsh northern winters [53], temperature and snow still affect them [11,15]. Deep snow requires more energy to walk through [16] and lowers movement rates [11]. Cold temperatures during mid-winter were not related to lower movement rates, in contrast with Eastland [11]; however, winter temperatures in the study area were relatively mild during this time period. Furthermore, the time scale (monthly) of my analysis is probably too coarse to detect these fine-scale patterns. The late winter movement rates reported here were very similar to those reported for the adjacent Fortymile Caribou Herd [24].

Mörschel and Klein [13] found that high temperatures had the greatest influence on caribou activity budgets during the summer months. I found that average temperature was positively correlated with movement rates throughout the year. Caribou movement rates were lower in months in which extreme temperatures (i.e., the average temperature deviated greatly from normal), both warmer and colder than normal, were encountered. This was especially true during migratory months (April, May, October, and November) and is consistent with other studies [54].

The NCH caribou that winter in the Tok area crossed the Richardson Highway, trans-Alaska pipeline, Tok (Cut-off) Highway, and Alaska Highway. There were no fatalities of instrumented caribou due to vehicular collisions related to these crossings. Survival of the more migratory caribou that wintered in the Tok area was not significantly different than either the CRB or Cantwell wintering caribou. This suggests that either (1) the costs and benefits of migration were balanced or (2) my ability to detect differences in survival of migratory caribou was difficult to detect due to limited sample size. Migration is an important behavioral trait that benefits large herds; however, increased vulnerability to mortality is as important a biological cost of migration for caribou as it is for other cervids [55,56]. The apparent lack of a clearly superior (migratory) strategy may select for multiple strategies within an individual population [42]. The increase in vulnerability of migratory individuals may be due to direct (e.g., increased exposure to predators) or indirect (e.g., additional energetic expense of migrating leading to caribou that are in poorer condition) causes. In the latter case, the proximate cause of the mortality might be predation, but the ultimate cause is poorer condition. Another consequence of poorer condition is reduced fecundity [57,58].

Caribou wearing GPS collars had a lower rate of survival than those with VHF collars. My data did not allow me to test if this was because the GPS collars were heavier (only by 0.3 kg), the fact that the GPS-collared caribou were captured up to six times during the project versus a single capture for VHF-collared caribou, or both. Regardless, this result should sound a cautionary note for researchers conducting repeated captures with radio-collared animals [59,60,61,62]. Even slight differences in collar weight can affect ungulate behavior and movement rates [63].

## 5. Conclusions

Long-distance migrations are an imperiled biological phenomenon [1]. Documenting migratory routes and understanding the diversity in migratory strategies are crucial first steps in managing and conserving these critical ecological processes [64]. Long-distance seasonal migrators, driven by resource availability [9], must balance costs and benefits. The majority of the NCH has typically wintered north of the Alaska Range for the past decade, where the biomass of preferred forage, fruticose lichens, is much greater [65]. I posit, with large herd sizes, the benefits of increased range quality are enough to offset the costs of excess energy requirements (as caribou are highly efficient walkers [9,16]) and increased risk of predation to reach this winter range. Alternative migratory strategies may be exhibited even within the same herd and are driven by environmental, demographic, and physiological factors. Some NCH caribou reside in smaller bands on peripheral ranges, have lower movement rates during much of the year, and migrate shorter distances than the rest of the herd. This alternative strategy may represent a bridge between highly migratory barren-ground and more sedentary woodland caribou.

## Figures and Tables

**Table 1:** Average (and standard deviation of) group size (number of caribou) by selected winter range (Cantwell, Tok, and Copper River Basin (CRB)) and month of the Nelchina Caribou Herd, Alaska, October 1999 September 2002.

Month	Cantwell	Tok	CRB
January	15 (8)	27 (39)	20 (13)
February	11 (7)	16 (15)	62 (72)
March	22 (16)	11 (8)	62 (86)
April	45 (56)	12 (11)	82 (101)
May	21 (14)	12 (10)	8 (5)
June	96 (157)	30 (27)	168 (171)
July	105 (146)	1543 (1655)	1925 (2281)
August	4 (2)	21 (70)	67 (125)
September	20 (21)	49 (55)	46 (83)
October	17 (13)	24 (25)	32 (57)
November	28 (65)	19 (18)	353 (604)
December	12 (8)	16 (27)	18 (14)

**Table 2:** Average movement rates (meters per hour) of adult, female caribou by selected Nelchina Caribou Herd winter range, east-central Alaska, October 1999 September 2002. CRB is Copper River Basin. An asterisk denotes differences in movement rates that are statistically significant.

Month	Tok	Cantwell	CRB	*F*	*d.f.*	*p*
January	112	81	158	3.20	2, 48	0.050 *
February	97	98	74	2.08	2, 45	0.138
March	90	85	88	0.09	2, 44	0.918
April	205	89	132	9.45	2, 49	0.000 *
May	356	189	273	7.35	2, 41	0.002 *
June	295	197	255	4.10	2, 39	0.025 *
July	446	283	412	5.77	2, 38	0.007 *
August	260	170	255	3.90	2, 36	0.030 *
September	371	263	370	4.23	2, 34	0.023 *
October	438	236	394	15.99	2, 50	0.000 *
November	274	143	222	7.02	2, 50	0.002 *
December	124	120	174	2.33	2, 50	0.108

**Table 3:** Models of factors related to the movement rates of adult, female caribou from the Nelchina Caribou Herd, Alaska, October 1999–September 2002. “WR” refers to the winter range that was utilized, “Ave. temp.” is average monthly temperature, “ABSADT” is the absolute value of the deviation from normal monthly temperature, “Group size” is the number of observed caribou, “Snow depth” is self-explanatory, and “Snow%ave” is the percentage that the snow depth is relative to average readings.

A. Year round			
**Model**	**AIC*_C_***	**Delta AIC*_C_***	**Model Weight**
Group size + Ave. temp. + ABSADT	2046.91	0.00	0.9867
Group size + WR + Ave. temp. + ABSADT	2055.67	8.76	0.0124
Ave. temp. + ABSADT	2062.19	15.28	0.0005
Group size + Ave. temp. + ABSADT	2062.36	15.45	0.0004
B. Summer (June, July, August, and September)			
**Model**	**AIC*_C_***	**Delta AIC*_C_***	**Model Weight**
Group size	1828.76	0.00	0.9976
Group size + WR	1842.34	13.58	0.0011
Group size. + ABSADT	1843.26	14.50	0.0007
Group size + Ave. temp.	1843.96	15.20	0.0005
C. Winter (December, January, February, and March)			
**Model**	**AIC*_C_***	**Delta AIC*_C_***	**Model Weight**
Ave. temp.	1984.61	0.00	0.9954
Group size + Ave. temp.	1996.17	11.56	0.0031
Ave. temp.+ ABSADT	1997.95	13.34	0.0013
Group size	2003.59	18.98	0.0001
D. Migration (April, May, October, and November)			
**Model**	**AIC*_C_***	**Delta AIC*_C_***	**Model Weight**
ABSADT	2046.91	0.00	0.9867
WR + ABSADT	2055.67	8.76	0.0124
Ave. temp.+ ABSADT	2062.19	15.28	0.0005
Group size + ABSADT	2062.36	15.45	0.0004
E. Late Winter (February, March, April, and May)			
**Model**	**AIC*_C_***	**Delta AIC*_C_***	**Model Weight**
Ave. temp. + Snow depth	1963.79	0.00	0.9874
Ave. temp. + Snow depth + Snow%ave	1972.87	9.08	0.0105
Ave. temp. + Snow depth + ABSADT	1976.96	13.17	0.0014
Group size + Ave. temp. + Snow depth	1978.29	14.50	0.0007
F. Mid-Winter (February and March)			
**Model**	**AIC*_C_***	**Delta AIC*_C_***	**Model Weight**
Group size	921.59	0	0.3509
Snow depth	922.95	1.36	0.1778
Ave. temp.	923.11	1.52	0.1641
Snow%ave	923.26	1.67	0.1523
ABSADT	923.27	1.68	0.1515
Group size + snow depth	933.66	12.07	0.0008

**Table 4:** Survival of adult, female caribou from the Nelchina Caribou Herd, Alaska, October 1999 September 2002. (A) The first model pools all caribou, whereas the second model divides caribou by selected winter range. (B) The first model divides caribou by collar type (VHF or GPS), whereas the second model pools all caribou. (C) The first model pools all caribou, whereas the second model divides caribou by selected winter range and collar type (VHF or GPS). # is used to denote number.

A.					
**Model**	**AIC*_C_***	**Delta AIC*_C_***	**Model Weight**	**# Parameters**	**Deviance**
All caribou the same	117.28	0.00	0.86283	1	18.328
Vary by wintering area	120.95	3.68	0.13717	3	17.943
B.					
**Model**	**AIC*_C_***	**Delta AIC*_C_***	**Model Weight**	**# Parameters**	**Deviance**
Vary by collar type	289.92	0.00	0.90348	2	65.840
All caribou the same	294.39	4.47	0.09652	1	72.317
C.					
**Model**	**AIC*_C_***	**Delta AIC*_C_***	**Model Weight**	**# Parameters**	**Deviance**
All caribou the same	123.68	0.00	0.65937	1	36.725
Vary by area and collar	125.00	1.32	0.34063	6	27.796

## Data Availability

The Nelchina Caribou Herd GPS data used in this study are available at: https://doi.org/10.5066/P1365EPK.
